# Supplementary material for: Extraction of High-Quality RNA from S. aureus Internalized by Endothelial Cells
Source: Microorganisms. 2023 Apr 13;11(4):1020. doi: 10.3390/microorganisms11041020 (PMC10143013; doi:10.3390/microorganisms11041020)
Supplement: Supplementary file 1 [file microorganisms-11-01020-s001.zip › microorganisms-2308337-supplementary.pdf]

Supplement to "Extraction of High-Quality RNA from *S. aureus* Internalized by Endothelial Cells"

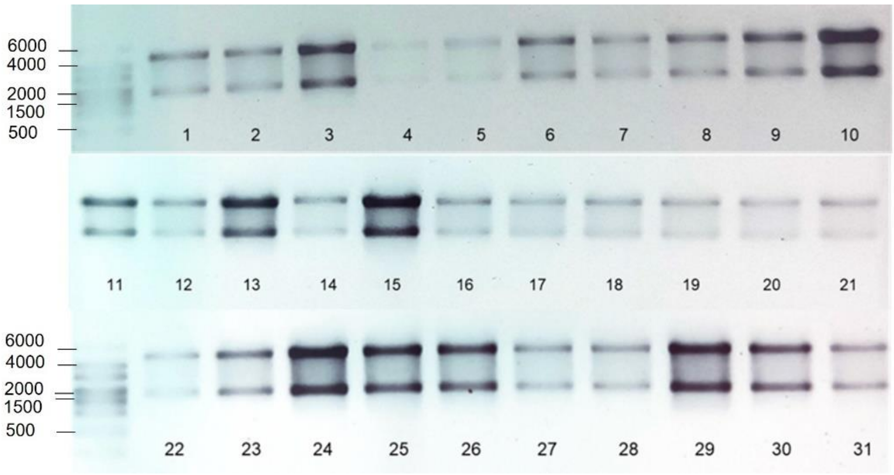

Supplementary Figure S1. Gel electrophoresis of endothelial RNA samples. RNA ladder in bp. Numbers refer to Supp. Table S1.

Supplementary Table S1: List of endothelial RNA control and infected samples. Numbers refer to Supp. Figure S1.

Control samples		*S. aureus* infected samples	
1	Non-infected 90 min p.i._1	16	Infected 90 min p.i._1
2	Non-infected 90 min p.i._2	17	Infected 90 min p.i._2
3	Non-infected 90 min p.i._3	18	Infected 90 min p.i._3
4	Non-infected 90 min p.i._4	19	Infected 90 min p.i._4
5	Non-infected 90 min p.i._5	20	Infected 90 min p.i._5
6	Non-infected 24 h p.i._1	21	Infected 90 min p.i._6
7	Non-infected 24 h p.i._2	22	Infected 24 h p.i._1
8	Non-infected 24 h p.i._3	23	Infected 24 h p.i._2
9	Non-infected 24 h p.i._4	24	Infected 24 h p.i._3
10	Non-infected 24 h p.i._5	25	Infected 24 h p.i._4
11	Non-infected 48 h p.i._1	26	Infected 24 h p.i._5
12	Non-infected 48 h p.i._2	27	Infected 48 h p.i._1
13	Non-infected 48 h p.i._3	28	Infected 48 h p.i._2
14	Non-infected 48 h p.i._4	29	Infected 48 h p.i._3
15	Non-infected 48 h p.i._5	30	Infected 48 h p.i._4
		31	Infected 48 h p.i._5

Supplementary Table S2: Overview of primers used in the study.

Endothelial primers (5'-3')	Forward	Reverse	Product size [bp]	Reference	Final concentration [nM]
GAPDH	GCAAATTTCCATGGCACCGT	GCCCCACTTGATTTTGGAGG	104	Tuchscherr et al., 2011 [28]	67
ACTB	AACTGGAACGGTGAAGGTG	CTGTGTGGACTTGGGAGAGG	209	Tuchscherr et al., 2011 [28]	67
ICAM1	ACCTCCCCACCCACATACATTT	GGCATAGCTTGGGCATATTCC	96	Tuchscherr et al., 2011 [28]	100
**Bacterial primers (5'-3')**	**Forward**	**Reverse**	**Product size [bp]**	**Reference**	**Final concentration [nM]**
gyrB	AATTGAAGCAGGCTATGTGT	ATAGACCATTTTGGTGTTGG	122	Tuchscherr et al., 2011 [28]	200
tmRNA	CACTCTGCATCGCCTAACAG	TCAAACGGCAGTGTTTAGCA	136	Bordeau et al., 2016 [32]	200
aroE	CTATCCACTTGCCATCTTTTAT	ATGGCTTTAATATCACAATTCC	83	Tuchscherr et al., 2011 [28]	200
gmk	AAGGTGCAAAGCAAGTTAGAA	CTTTACGCGCTTCGTTAATAC	150	Tuchscherr et al., 2011 [28]	200
hu	CCTCAAAGTTACCGAAACCAA	AGCTGGTTCAGCAGTAGATGC	95	Buvelot et al., 2021 [33]	200
agrA	AACTGCACATACACGCTTACA	GGCAATGAGTCTGTGAGATTT	155	Tuchscherr et al., 2011 [28]	200
eap	TGCATATGGAACATGGACTTTAGAA	AAGCGTCTGCCGCAGCTA	83	Tuchscherr et al., 2011 [28]	200
fnbA	ACAAGTTGAAGTGGCACAGCC	CCGCTACATCTGCTGATCTTGTC	74	Vaudaux et al., 2002 [34]	125

Supplementary Table S3: Ranking of *S. aureus* housekeeping genes according to their transcript length and summary of ranking in different conditions.

Ranking	Housekeeping gene	Tanscript lenght [bp]	Condition I: *S. aureus* from culture	Condition II: intracellular *S. aureus*	Across both conditions
1	*gyrB*	1935	4	1	1
2	*aroE*	802	5	5	4
3	*gmk*	624	1	4	3
4	*tmRNA*	362	2	3	1
5	*hu*	273	3	2	2
